# Supplementary material for: Micropropagation and Acclimatization of Gymnocalycium cv. Fancy (Cactaceae): Developmental Responses to Different Explant Types and Hormone Conditions
Source: Plants (Basel). 2023 Nov 22;12(23):3932. doi: 10.3390/plants12233932 (PMC10708245; doi:10.3390/plants12233932)
Supplement: Supplementary file 1 [file plants-12-03932-s001.zip › plants-2684718-supplementary.pdf]

Supplementary Table S1. Average numbers with their standard errors and total number of calluses obtained monthly per condition.

	INDUCTION PERIOD IN PRESENCE OF PGRs						DEVELOPMENT PERIOD IN ABSENCE OF PGRs					
	**1st MONTH**		**2nd MONTH**		**3rd MONTH**		**4th MONTH**		**5th MONTH**		**6th MONTH**	
**Condition (1)**	**Average**	**Nº of callus**	**Average**	**Nº of callus**	**Average**	**Nº of callus**	**Average**	**Nº of callus**	**Average**	**Nº of callus**	**Average**	**Nº of callus**
Control _A	0.00 ± 0.00	0	0.00 ± 0.00	0	0.00 ± 0.00	0	0.00 ± 0.00	0	0.00 ± 0.00	0	0.00 ± 0.00	0
Control_B	0.00 ± 0.00	0	0.00 ± 0.00	0	0.00 ± 0.00	0	0.06 ± 0.06	1	0.06 ± 0.06	1	0.00 ± 0.00	0
Control_DC	0.00 ± 0.00	0	0.00 ± 0.00	0	0.00 ± 0.00	0	0.00 ± 0.00	0	0.00 ± 0.00	0	0.00 ± 0.00	0
BAP2_A	0.00 ± 0.00	0	0.06 ± 0.06	1	0.00 ± 0.00	0	0.00 ± 0.00	0	0.00 ± 0.00	0	0.00 ± 0.00	0
BAP2_B	0.00 ± 0.00	0	0.00 ± 0.00	0	0.00 ± 0.00	0	0.00 ± 0.00	0	0.06 ± 0.06	1	0.00 ± 0.00	0
BAP2_CD	0.00 ± 0.00	0	0.38 ± 0.13	6	0.06 ± 0.06	1	0.06 ± 0.06	1	0.00 ± 0.00	0	0.00 ± 0.00	0
BAP4_A	0.00 ± 0.00	0	0.00 ± 0.00	0	0.00 ± 0.00	0	0.00 ± 0.00	0	0.00 ± 0.00	0	0.00 ± 0.00	0
BAP4_B	0.00 ± 0.00	0	0.00 ± 0.00	0	0.19 ± 0.10	3	0.13 ± 0.13	2	0.00 ± 0.00	0	0.00 ± 0.00	0
BAP4_CD	0.00 ± 0.00	0	0.00 ± 0.00	0	0.00 ± 0.00	0	0.00 ± 0.00	0	0.00 ± 0.00	0	0.00 ± 0.00	0
BAP8_A	0.19 ± 0.14	3	0.13 ± 0.09	2	0.06 ± 0.06	1	0.00 ± 0.00	0	0.00 ± 0.00	0	0.00 ± 0.00	0
BAP8_B	0.00 ± 0.00	0	0.31 ± 0.15	5	0.25 ± 0.17	4	0.13 ± 0.13	2	0.00 ± 0.00	0	0.00 ± 0.00	0
BAP8_CD	0.13 ± 0.09	2	0.13 ± 0.13	2	0.06 ± 0.06	1	0.00 ± 0.00	0	0.00 ± 0.00	0	0.00 ± 0.00	0
KIN2_A	0.00 ± 0.00	0	0.00 ± 0.00	0	0.00 ± 0.00	0	0.00 ± 0.00	0	0.00 ± 0.00	0	0.00 ± 0.00	0
KIN2_B	0.00 ± 0.00	0	0.00 ± 0.00	0	0.13 ± 0.13	2	0.13 ± 0.13	2	0.25 ± 0.14	4	0.25 ± 0.14	4
KIN2_CD	0.00 ± 0.00	0	0.00 ± 0.00	0	0.06 ± 0.06	1	0.00 ± 0.00	0	0.00 ± 0.00	0	0.00 ± 0.00	0
KIN4_A	0.00 ± 0.00	0	0.00 ± 0.00	0	0.00 ± 0.00	0	0.00 ± 0.00	0	0.00 ± 0.00	0	0.00 ± 0.00	0
KIN4_B	0.00 ± 0.00	0	0.00 ± 0.00	0	0.06 ± 0.06	1	0.13 ± 0.13	2	0.25 ± 0.17	4	0.25 ± 0.17	4
KIN4_CD	0.00 ± 0.00	0	0.00 ± 0.00	0	0.00 ± 0.00	0	0.13 ± 0.13	2	0.31 ± 0.15	5	0.31 ± 0.15	5
KIN8_A	0.00 ± 0.00	0	0.00 ± 0.00	0	0.00 ± 0.00	0	0.00 ± 0.00	0	0.00 ± 0.00	0	0.00 ± 0.00	0
KIN8_B	0.00 ± 0.00	0	0.13 ± 0.13	2	0.13 ± 0.13	2	0.13 ± 0.09	2	0.13 ± 0.09	2	0.13 ± 0.09	2
KIN8_CD	0.00 ± 0.00	0	0.07 ± 0.07	1	0.00 ± 0.00	0	0.00 ± 0.00	0	0.00 ± 0.00	0	0.00 ± 0.00	0
TDZ1_A	1.44 ± 0.36 a*	23	1.88 ± 0.38 a*	30	2.00 ± 0.37 a*	32	1.49 ± 0.32 a*	25	1.31 ± 0.36 ab*	21	1.13 ± 0.29 ab*	18
TDZ1_B	0.13 ± 0.09 c	2	0.69 ± 0.22 cd*	11	1.06 ± 0.23 ab*	17	0.75 ± 0.26 ab*	12	0.44 ± 0.16 ab*	7	0.31 ± 0.15 b	5
TDZ1_CD	0.19 ± 0.10 c	3	0.75 ± 0.28 cd*	12	0.75 ± 0.25 ab*	12	0.69 ± 0.21 c*	11	0.44 ± 0.22 ab*	7	0.44 ± 0.22 ab*	7
TDZ2_A	0.69 ± 0.20 b*	11	1.19 ± 0.21 ab*	19	1.13 ± 0.29 ab*	18	1.13 ± 0.27 ab*	18	1.19 ± 0.42 ab*	19	1.19 ± 0.42 ab*	19
TDZ2_B	0.13 ± 0.09 c	2	0.44 ± 0.20 cd*	7	0.69 ± 0.22 c*	11	0.50 ± 0.18 c*	8	0.19 ± 0.10 b	3	0.19 ± 0.10 b	3
TDZ2_CD	0.06 ± 0.06 c	1	1.13 ± 0.26 ab*	18	1.00 ± 0.26 ab*	16	0.69 ± 0.22 ab*	11	0.31 ± 0.15 b	5	0.31 ± 0.15 b	5
TDZ4_A	0.75 ± 0.21 b*	12	1.00 ± 0.27 abc*	16	1.38 ± 0.30 ab*	22	1.38 ± 0.30 ab*	22	1.44 ± 0.34 a*	23	1.38 ± 0.30 a*	22
TDZ4_B	0.00 ± 0.00 c	0	0.56 ± 0.20 cd*	9	0.94 ± 0.25 ab*	15	0.81 ± 0.25 ab*	13	0.81 ± 0.25 ab*	13	0.75 ± 0.21 ab*	12
TDZ4_CD	0.00 ± 0.00 c	0	0.13 ± 0.09 d	2	0.50 ± 0.16 c*	8	0.63 ± 0.24 c*	10	0.63 ± 0.24 ab*	10	0.63 ± 0.24 ab*	10
**TOTAL CALLUS**		**59**		**143**		**167**		**144**		**125**		**116**

[1] Conditions: CONTROL (control group), BAP (6-Benzylaminopurine), KIN (Kinetin) and TDZ (Thidiazuron). The numbers following the conditions indicate the hormonal concentration (1, 2, 4 or 8 μM) to which the explants have been subjected. Capital letters indicates the explants used in each combination: A (apices), B (bases) and CD (central discs).

* Results different from 0 according to Student-Newman-Keuls 95% analysis. Values followed by the same letter are not statistically different for P =0.05 according to Student-Newman-Keuls.

Supplementary Table S2. Average shoot productivity and the significance of the factors and their interactions per month.

		INDUCTION PERIOD IN PRESENCE PGRs						DEVELOPMENT PERIOD IN ABSENCE OF PGRs					
		1st MONTH		2nd MONTH		3rd MONTH		4th MONTH		5th MONTH		6th MONTH	
**PRODUCTIVITY [(1)]**	**CASES**	**P-Value**	**Average [(3)]**	**P-Value**	**Average [(3)]**	**P-Value**	**Average [(3)]**	**P-Value**	**Average [(3)]**	**P-Value**	**Average [(3)]**	**P-Value**	**Average [(3)]**
**H**		0.004*		0.001*		0.004*		0.802		0.083		0.000*	
BAP	192		0.026 ± 0.019 a		0.417 ± 0.059 b		0.656 ± 0.076 b		0.938 ± 0.090 a		1.297 ± 0.127 a		1,359 ± 0,129 a
KIN	192		0.000 ± 0.000 a		0.277 ± 0.048 a		0.586 ± 0.068 b		0.853 ± 0.076 a		1.126 ± 0.095 a		1,220 ± 0,104 a
TDZ	192		0.010 ± 0.007 a		0.182 ± 0.044 a		0.406 ± 0.065 a		0.833 ± 0.070 a		1.411 ± 0.107 a		2,271 ± 0,130 b
**TE**		0.000*		0.000*		0.000*		0.000*		0.000*		0.000*	
Apices	192		0.010 ± 0.007 a		0.104 ± 0.036 a		0.156 ± 0.047 a		0.286 ± 0.053 a		0.453 ± 0.076 a		0,807 ± 0,110 a
Bases	192		0.000 ± 0.000 a		0.250 ± 0.048 b		0.510 ± 0.068 b		0.964 ± 0.080 b		1.448 ± 0.122 b		1,875 ± 0,139 b
Central Discs	192		0.026 ± 0.019 a		0.524 ± 0.062 c		0.984 ± 0.079 c		1.377 ± 0.081 c		1.937 ± 0.101 c		2,173 ± 0,106 c
**HC [(2)]**		0.410		0.882		0.410		0.005*		0.004*		0.000*	
Control	144		0.000 ± 0.000 a		0.264 ± 0.055 a		0.493 ± 0.072 a		0.743 ± 0.089 a		1.021 ± 0.122 a		1,125 ± 0,130 a
Low	144		0.035 ± 0.025 a		0.333 ± 0.067 a		0.576 ± 0.086 a		0.924 ± 0.094 ab		1.424 ± 0.134 b		1,764 ± 0,148 b
Medium	144		0.007 ± 0.007 a		0.271 ± 0.053 a		0.632 ± 0.083 a		1.042 ± 0.086 b		1.431 ± 0.113 b		1,833 ± 0,135 b
High	144		0.007 ± 0.007 a		0.301 ± 0.061 a		0.497 ± 0.082 a		0.790 ± 0.096 a		1.238 ± 0.141 ab		1,748 ± 0,163 b
**H x TE**		0.043*		0.000*		0.000*		0.000*		0.000*		0.000*	
**H x HC**		0.056		0.335		0.226		0.117		0.003*		0.000*	
**TE x HC**		0.056		0.007*		0.000*		0.050*		0.053		0.002*	
**H x TE x HC**		0.075		0.213		0.033*		0.171		0.176		0.063	
**TOTAL CASES**	**576**												
**TOTAL AVERAGE**			**0.012 ± 0.007**		**0.292 ± 0.030**		**0.550 ± 0.040**			**0.875 ± 0.046**		**1.278 ± 0.064**	

[1] Productivity depending on factors: H=Hormones; TE=Type of Explant; HC=Hormone Concentration; BAP=6-Benzylaminopurine; KIN= Kinetin; TDZ= Thidiazuron. PGRs= Plant Growth Regulators.

[2] Control= In absence of PGRs; Low = Corresponds to the concentrations of BAP2 (2 µM), KIN2 (2 µM), TDZ1 (1 µM); Medium = Corresponds to the concentrations of BAP4 (4 µM), KIN4 (4 µM), TDZ2 (2 µM); High = Corresponding to the concentrations of BAP8 (8 µM), KIN8 (8 µM), TDZ4 (4 µM).

[3] Averages with their standard erros. Values followed by different letters are statistically different for P =0.05 according to Student-Newman-Keuls.

* Significant results at P≤ 0.05

Supplementary Table S3. Average shoot efficiency and the significance of the factors and their interactions per month.

		INDUCTION PERIOD IN PRESENCE PGRs						DEVELOPMENT PERIOD IN ABSENCE OF PGRs					
		1st MONTH		2nd MONTH		3rd MONTH		4th MONTH		5th MONTH		6th MONTH	
**EFFICIENCY [(1)]**	**CASES**	**P-Value**	**Average [(3)]**	**P-Value**	**Average [(3)]**	**P-Value**	**Average [(3)]**	**P-Value**	**Average [(3)]**	**P-Value**	**Average [(3)]**	**P-Value**	**Average [(3)]**
**H**		0.228		0.000*		0.000*		0.059				0.000*	
BAP	192		0.599 ± 0.442 a		6.694 ± 0.966 b		10.402 ± 1.233 b		14.472 ± 1.423 ab	0.489	19.707 ± 1.923 a		20.589 ± 2.004 a
KIN	192		0.000 ± 0.000 a		6.345 ± 1.198 b		12.334 ± 1.651 b		16.856 ± 1.785 b		21.690 ± 2.123 a		23.001 ± 2.214 a
TDZ	192		0.065 ± 0.046 a		2.450 ± 0.689 a		6.487 ± 1.151 a		11.955 ± 1.291 a		20.051 ± 1.926 a		29.054 ± 2.046 b
**TE**		0.228		0.000*		0.000*		0.000*		0.000*		0.000*	
Apices	192		0.065 ± 0.046 a		0.649 ± 0.229 a		0.993 ± 0.300 a		1.817 ± 0.336 a		2.819 ± 0.475 a		5.094 ± 0.705 a
Bases	192		0.000 ± 0.000 a		2.171 ± 0.461 a		4.336 ± 0.604 b		8.040 ± 0.699 b		11.942 ± 1.023 b		15.270 ± 1.156 b
Central Discs	192		0.602 ± 0.444 a		12.703 ± 1.482 b		23.955 ± 1.900 c		33.514 ± 1.843 c		46.819 ± 2.244 c		52.433 ± 2.234 c
**HC [(2)]**		0.198		0.743		0.081		0.001*		0.001*		0.000*	
Control	144		0.000 ± 0.000 a		5.223 ± 1.159 a		9.361 ± 1.453 a		12.339 ± 1.653 a		16.745 ± 2.131 a		18.310 ± 2.265 a
Low	144		0.799 ± 0.589 a		6.140 ± 1.309 a		11.158 ± 1.837 a		15.662 ± 1.940 ab		22.941 ± 2.518 bc		27.145 ± 2.641 b
Medium	144		0.046 ± 0.046 a		5.137 ± 1.108 a		11.510 ± 1.729 a		17.632 ± 1.882 b		23.985 ± 2.364 c		27.990 ± 2.488 b
High	144		0.041 ± 0.041 a		4.137 ± 0.925 a		6.897 ± 1.221 a		12.044 ± 1.463 a		18.236 ± 2.119 ab		23.417 ± 2.229 b
**H x TE**		0.057		0.000*		0.000*		0.000*		0.000*		0.000*	
**H x HC**		0.051		0.318		0.301		0.564		0.079		0.019*	
**TE x HC**		0.051		0.017*		0.000*		0.043*		0.043*		0.027*	
**H x TE x HC**		0.040*		0.449		0.407		0.206		0.515		0.266	
**TOTAL CASES**	**576**												
**TOTAL AVERAGE**			**0.222 ± 0.148**		**5.161 ± 0.566**		**9.737 ± 0.791**		**14.424 ± 0.876**		**20.481 ± 1.149**		**24.217 ± 1.213**

[1] Efficiency depending on factors: H=Hormones; TE=Type of Explant; HC=Hormone Concentration; BAP=6-Benzylaminopurine; KIN= Kinetin; TDZ= Thidiazuron. PGRs= Plant Growth Regulators.

[2] Control= In absence of PGRs; Low = Corresponds to the concentrations of BAP2 (2 µM), KIN2 (2 µM), TDZ1 (1 µM); Medium = Corresponds to the concentrations of BAP4 (4 µM), KIN4 (4 µM), TDZ2 (2 µM); High = Corresponding to the concentrations of BAP8 (8 µM), KIN8 (8 µM), TDZ4 (4 µM).

[3] Averages with their standard erros. Values followed by different letters are statistically different for P =0.05 according to Student-Newman-Keuls.

* Significant results at $P \leq 0.05$
